# Integrated bioinformatics-based identification of proliferative diabetic retinopathy and idiopathic pulmonary fibrosis: Focus on fibrosis and immune infiltration

**DOI:** 10.1371/journal.pone.0343398

**Published:** 2026-02-23

**Authors:** Juanhui Cao, Fangyuan Dong, Xianfeng Li, Xuechun Zhu

**Affiliations:** 1 Department of Ophthalmology, Changsha Central Hospital, Changsha Central Hospital Affiliated to University of South China, Changsha, China; 2 Department of laboratory, Hunan Aerospace Hospital, Changsha, China; Chuo University, JAPAN

## Abstract

Proliferative diabetic retinopathy (PDR) and idiopathic pulmonary fibrosis (IPF) share the common pathological feature of fibrosis, but the connecting mechanisms are not well – understood. This study aimed to identify potential shared genes and therapeutic drug candidates for fibrosis in both conditions by analyzing transcriptome datasets from the Gene Expression Omnibus (GEO). Differential expression analysis of two training datasets revealed 90 fibrosis – related genes. These genes were then analyzed for gene function enrichment, protein - protein interaction (PPI), transcription factor (TF) network, immune cell infiltration, and drug prediction. The findings underscored the role of immune activation in fibrosis progression. Among the 13 identified hub genes, however, six demonstrated strong correlations and high diagnostic potential upon further validation. The study also pinpointed six drug molecules with significant enrichment values, three of which showed promising results in blind docking simulations. These six genes and three drug molecules serve as potential targets for diagnosing and treating fibrosis in both PDR and IPF, though additional research is necessary to confirm their clinical utility.

## Introduction

Diabetic retinopathy (DR) has emerged as a leading cause of permanent vision loss among adults of working age globally [1–3]. Proliferative diabetic retinopathy (PDR), the most severe and treatment-resistant stage of diabetic retinopathy, is marked by pathological angiogenesis and uncontrolled cellular proliferation [4]. Epidemiological data suggest that approximately 1.4% of diabetic patients worldwide develop this vision-threatening complication [5]. For now, the clinical management of PDR remains a significant challenge in ophthalmology [6,7]. Cutting-edge omics technologies and computational biology approaches have substantially enhanced our understanding of PDR pathogenesis, enabling more targeted therapeutic development [8]. Consequently, identifying new molecular markers and developing innovative intervention strategies for PDR is essential to improve patient outcomes.

Idiopathic pulmonary fibrosis (IPF) is a chronic and progressive interstitial lung disease characterized by irreversible fibrosis and significant damage to the lung parenchyma [9]. Epidemiological data show significant geographic differences in disease burden. Reported prevalence rates range from 3–25 cases per 100,000 people in European nations and 6–45 in Asian populations. In contrast, North American cohorts show higher frequencies of 24–30 cases per 100,000 individuals. The disease has a very poor prognosis. Median survival after diagnosis is generally only 24–60 months due to the relentless and treatment-resistant progression of the disease [10,11]. The evident pathological hallmark of IPF is relentless fibrotic remodeling in the pulmonary interstitium [12]. Currently, therapeutic approaches mainly focus on disease modification rather than a cure, and antifibrotic pharmacotherapy is the key in clinical management [13]. These targeted pharmacological strategies aim to slow down functional deterioration and disease progression.

Proliferative diabetic retinopathy (PDR) and idiopathic pulmonary fibrosis (IPF) represent two clinically significant diseases with distinct yet interconnected pathological profiles. PDR and IPF are both fibrotic diseases, and they share common pathophysiological features involving abnormal angiogenesis and regression, uncontrolled deposition of ECM, and immune microenvironment dysregulation. Emerging evidence highlights a profound connection between PDR and IPF, both featuring relentless fibrotic remodeling and heightened immune activation during their progression [14,15]. Mitigating fibrotic advancement and modulating inflammatory cascades are of great importance for improving patient prognoses in these conditions [16]. This study aims to elucidate the intricate characteristics of fibrotic transformation and immune stress crosstalk in PDR and IPF. Thereby, it offers actionable insights to refine therapeutic strategies.

Recent research has highlighted PDR and IPF as clinically significant fibroproliferative disorders with interconnected pathological narratives. Moreover, epidemiological evidence shows pathophysiological links between these conditions. Both exhibit severe fibrotic remodeling and uncontrolled immune dysregulation during disease progression. New therapeutic strategies aimed at interrupting fibrotic cascades and regulating immune pathways could potentially change clinical courses for PDR and IPF cases [17]. Therefore, we led this study to analyzes the common fibrotic features and similar immunopathological aspects of PDR and IPF.

We conducted bioinformatics research based on transcriptomic data from public databases and used differential expression genes (DEGs) analysis to identify fibrosis-related genes [18]. We conducted systematic analyses, including gene set enrichment analysis (GSEA), Gene Ontology (GO), Kyoto Encyclopedia of Genes and Genomes (KEGG) analysis, and immune cell analysis, to elucidate the biological mechanisms of biomarkers in PDR and IPF progression. We utilized protein-protein interaction network analysis to pinpoint hub genes. We used two independent validation set to verify the diagnostic ability of hub genes [19]. Additionally, we used the Connectivity Map (cMAP) database and Autodock software to explore small molecule drugs with potential therapeutic effects on fibrosis progression in both diseases [20].

In summary, our investigation reveals the common molecular mechanisms underlying PDR and IPF about fibrosis. The discovery of key regulatory genes and promising drug candidates could enable novel diagnostic approaches and therapeutic interventions for both conditions. Additional rigorous experimental validation is necessary.

## Materials and methods

### Microarray datasets acquisition

In our study on pertinent diseases, we implemented specific data collection and processing procedures. Gene expression datasets associated with PDR and IPF were systematically obtained from the Gene Expression Omnibus (GEO) repository. The data were retained for further analysis only when they met the following criteria: (1) The study type was restricted to expression profiling via array. (2) The sample was derived from *Homo sapiens*. (3) Information regarding the technology and platform of the study was furnished. (4) The study was publicly published and accessible. Finally, the microarray datasets GSE94019, GSE10667, GSE102485, and GSE24206 were incorporated into our study. For the training phase, we selected the series GSE94019 that included 9 PDR patients and 4 non-diabetic control subjects, and the dataset GSE10667 that included 8 IPF cases and 15 normal samples. As detailed in Table 1, validation analyses were performed using the dataset GSE102485 (22 PDR vs 3 control samples) and GSE24206 (17 IPF cases vs 6 healthy controls). Details of the samples that we used for the subsequent analysis in these datasets are presented in Table 1. We conducted a comprehensive search of the GeneCards database (https://www.genecards.org/) and identified 11,089 genes associated with fibrosis for further analysis (S1 Table).

**Table 1:** Provides a detailed overview of the research datasets utilized in this study.

Dataset	Publication	Platform	Classification	Application
GSE94019	Diabetes	GPL11154 Illumina HiSeq 2000 (*Homo sapiens*)	PRD:9, CC:4	Analysis
GSE10667	Am J Respir Crit Care Med	GPL4133 Agilent-014850 Whole Human Genome Microarray 4x44K G4112F (Feature Number version)	IPF:8, CC:15	Analysis
GSE102485	Invest Ophthalmol Vis Sci	GPL18573 Illumina NextSeq 500 (*Homo sapiens*)	PDR:22, CC:3	Validation
GSE24206	Am J Physiol Lung Cell Mol Physiol	GPL570 [HG-U133_Plus_2] Affymetrix Human Genome U133 Plus 2.0 Array	IPF:17, CC:6	Validation

PDR, Proliferative diabetic retinopathy; IPF, Idiopathic pulmonary fibrosis; CC, normal sample.

### Data preprocessing

Obtain microarray data from Series Matrix files and normalize them using the robust multiarray average (RMA) algorithm to remove potential batch effects [21]. This preprocessing step was conducted using R (version 4.2.1).

### DEG analysis

Differential gene expression analysis was performed utilizing the limma package (version 3.52.2) of R (version 4.2.1). Gene selection criteria included an adjusted *p*-value below 0.05 and an absolute log_2_ fold change exceeding 1.0. Then, we visualized differentially expressed genes using volcano plots from the ggplot2 package (version3.4.4). Additionally, based on the previous analysis, we identified and visualized common differentially expressed genes across multiple comparisons using Venn diagram analysis from ggplot2 and VennDiagram packages (version 1.7.3) in R. Finally, We used the ComplexHeatmap (version 2.13.1) package of R programming environment to visualize the data distribution.

### Functional classification and pathway enrichment

Gene set enrichment analysis (GSEA) is a method used to determine whether a predefined set of genes shows statistically significant differences in expression under various conditions. To investigate the biological functions of all genes in the progression of PDR and IPF, we conducted GSEA between the control and PDR or IPF groups by using clusterProfiler package (version 4.4.4) and msigdbr package of R. An adjusted *p*-value threshold of <0.05 was employed to determine statistical significance. Bubble charts were created to visualize the results using the ggplot2 package (version 3.4.4) in R.

Using the clusterProfiler package and org.Hs.e.g.,db package of R, we conducted Gene Ontology (GO), Kyoto Encyclopedia of Genes and Genomes (KEGG) analysis pathway enrichment analyses for DEGs2 in order to clarify the underlying biological mechanisms. The results were visualized using bubble charts with the ggplot2 package of R.

### Development of protein-protein interaction networks

Protein-protein interaction (PPI) networks are computational models. They delineate molecular interactions among gene products at the proteomic level, which helps to understand disease mechanisms and identify therapeutic targets [22]. The PPI networks for differentially expressed genes were created with the STRING database (version 11.5) with a strict interaction threshold (combined score >0.4) [23]. The network data were imported into Cytoscape (version 3.10.3) for visualization and topological analysis. To identify hub genes, we utilized the maximal clique centrality (MCC) algorithm for ranking network nodes and incorporated three Cytoscape plugins: CytoMcode, CytoNCA, and CytoHubba [24]. Then the top 15 genes from each analytical method were analyzed using Venn diagram analysis implemented in the from ggplot2 and VennDiagram packages in R to find their intersection and determine consensus hub genes.

### Therapeutic small molecule screening and characterization

Potential therapeutic compounds were screened from the Connectivity Map (cMAP) database of the Broad Institute. To identify antifibrotic agents effective for both PDR and IPF, we examined the overlap between differentially expressed genes (DEGs) from the GSE94019 and GSE10667 datasets and fibrosis-associated genes. Compounds demonstrating strong negative correlation scores in cMAP analysis were prioritized as promising candidates. Subsequently, structural and pharmacological data for these molecules were retrieved. The data were obtained from PubChem and the RCSB Protein Data Bank for further characterization.

### Validation of hub genes expression

We validated the reliability of the identified hub genes by analyzing their expression patterns in two separate datasets, GSE102485 and GSE24206. To compare gene expression profiles between pathological conditions (PDR or IPF) and their respective control groups (non-DR or non-IPF), we executed all data analyses and visualizations using the ggplot2, stats (4.2.1) and car (3.1−0) packages in the R statistical computing environment. Intergroup comparisons were made using the Mann-Whitney U test. Statistical significance was considered when the *P*-value was less than 0.05.

### Diagnostic performance assessment of key biomarkers using ROC analysis

We assessed the diagnostic predictive ability of the core genes through receiver operating characteristic (ROC) curve analysis. The discriminatory power of these biomarkers was quantified by calculating the area under the ROC curve (*AUC*) using a validated online analytical platform. According to established criteria, diagnostic performance was classified as non-informative (*AUC* ≤ 0.5), moderately informative (0.5 < *AUC* < 0.7), and highly informative (*AUC* ≥ 0.7).

### Comprehensive profiling of Immune cell infiltration patterns in disease pathogenesis

The Gene Set Variation Analysis (GSVA) algorithm (version 1.44.5) was applied to systematically evaluate immune cell infiltration in PDR, IPF, and healthy control groups. Specifically, a curated collection of immune cell-specific marker genes, obtained from seminal studies by Bindea et al. and Senbabaoglu et al., was used for single-sample Gene Set Enrichment Analysis (ssGSEA). Infiltration scores for various immune cell subsets were calculated using the “gsva” package (1.44.5) in R software, following the standard parameters recommended by the package documentation.

Subsequently, to validate the robustness of our findings, we replicated the immune cell infiltration analysis in two independent validation cohorts. In these cohorts, the Mann-Whitney U test was performed to compare the infiltration scores of 24 immune cell subsets between the disease groups (PDR and IPF) and the healthy control group. Within these same validation cohorts, we further assessed the associations (using Spearman correlation analysis) between immune cell infiltration and six key genes identified in our preceding analysis. Heatmap visualization of the infiltration patterns was generated using the “ComplexHeatmap” package in R, with samples clustered based on Euclidean distance, and complete linkage to highlight intergroup differences. A two-tailed *p*-value less than 0.05 was considered statistically significant for all analyses.

### Construction of transcriptional regulatory networks

The hub genes were analyzed using NetworkAnalyst (v3.0) to construct transcription factor-gene interaction and TF-miRNA co-regulatory networks. For TF-gene interaction and TF-miRNA co-regulatory networks construction, binding site predictions came from the JASPAR database and RegNetwork repository. The RegNetwork repository consolidates known regulatory interactions from multiple sources along with computationally predicted interactions based on transcription factor binding motifs. Network visualization and topological analysis were performed through the NetworkAnalyst computational platform.

### Identification of candidate drugs

Drug molecules were predicted using the cMAP (Connectivity MAP) based on the identified hub DEGs for PDR and IPF. Molecular docking simulations using Autodock4 (v4.2.6) were conducted to predict the interaction between the protein’s active site and the ligand. Pymol software (version 3.1.6) was utilized to derive affinity parameters and 3D structures from the binding energy data.

## Results

### Genes co-expressed in PDR, IPF, and fibrosis

**Fig 1:**
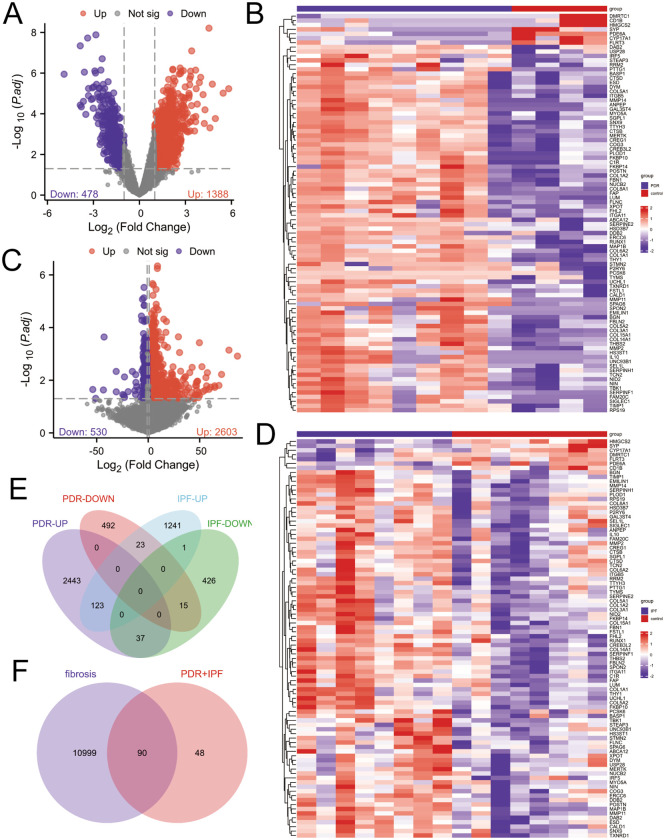
Volcano plot, venn diagram and heatmaps for identifying DEGs. **(A)** Volcano plot of DEGs in GSE10667 includes two vertical dashed lines indicating *log*_*2*_*FC* values of −1 and 1. **(B)** Heatmap of DEGs2 in GSE94019. In the study, red, blue, and white colors indicate upregulated DEGs2, downregulated DEGs2, and no significant changes, respectively. **(C)** Presents a volcano plot of DEGs in GSE94019. **(D)** Shows a Venn diagram (DEGs1) comparing DEGs between two datasets. **(E)** Displays a heatmap of DEGs2 in GSE10667. **(F)** Illustrates a Venn diagram (DEGs2) comparing DEGs1 with fibrosis-related datasets.

S1 Fig shows the study flowchart. Genes with *P <* 0.05 and |*log*_*2*_*FC*| > 1.0 were classified as DEGs by Student’s t-test. In total, 3133 DEGs (2603 upregulated and 530 downregulated genes) were identified in the GSE94019 dataset (S2 Table), and 1865 DEGs (1387 upregulated and 478 downregulated genes) were identified in the GSE10667 dataset (S3 Table). Volcano plots in Fig 1A and 1C separately illustrated these findings. Subsequently, we identified co-expressed genes shared by both datasets (S4 Table). Using Venn diagrams (Fig 1D), we screened 123 upregulated and 15 downregulated genes as potential key genes. This finding suggests a shared pathogenesis between PDR and IPF. We defined these potential crosstalk genes as DEGs1. To further identify DEGs2, we intersected 11089 fibrosis-related genes with DEGs1. A total of 90 DEGs were found, with 85 upregulated and 5 downregulated in PDR and IPF (S5 Table). We defined fibrosis-related DEGs as DEGs2 (Fig 1F). The heatmap illustrates that the relative gene expression of DEGs2 varies between the PDR, IPF and control groups (Fig 1B and 1E).

### Functional enrichment analysis

**Fig 2:**
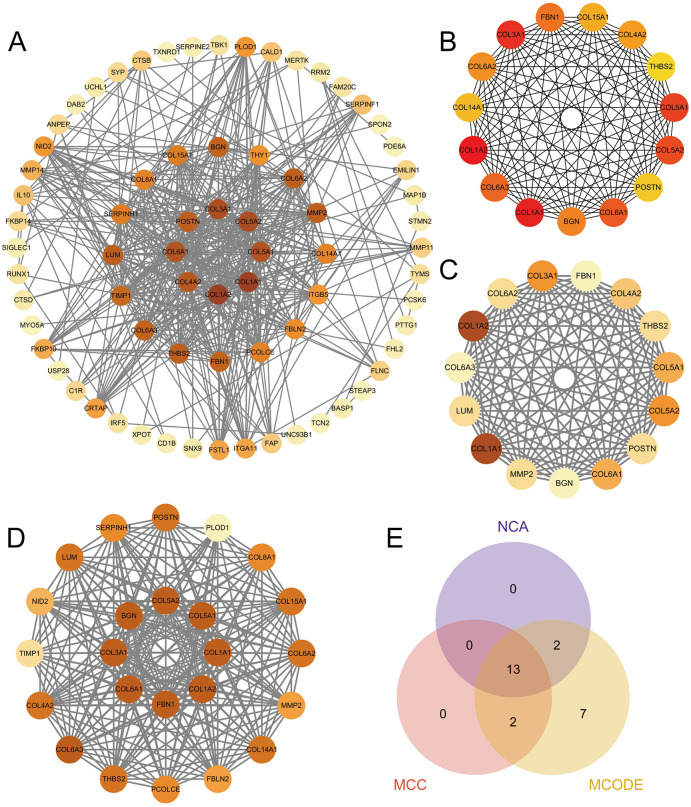
Identification of hub genes by using cytoscape. **(A)** Visualized PPI networks using Cytoscape software; **(B)** Identified hub genes from the PPI network with the CytoHubba plug-in based on MCC algorithms; **(C)** Selected potential hub genes using the NCA plug-in by degree without weight; **(D)** Determined hub genes from the PPI network with the MCODE plug-in; **(E)** Used Venn diagrams to find the intersection of genes from the three methods, selecting thirteen hub genes.

MSigDB’s gene set enrichment analysis (GSEA) identified significant perturbations in curated gene sets. The bubble chart of GSEA depicted in S2 Fig lists the top 10 gene sets. The analysis of GSE94019 revealed significant enrichment in the following pathways: Diseases of Signal Transduction by Growth Factor Receptors and Second Messengers (*p*.adj = 2.85E-03), VEGFA-VEGFR2 Signaling Pathway (*p*.adj = 2.85E-06), *RHO* GTPase Cycle (*p*.adj = 2.37E-04), and Regulation of Actin Cytoskeleton (*p*.adj = 1.61E-05) (S2 Fig 2A). In addition, the Cell Cycle Mitotic(*p*.adj = 4.37E-07), Degradation of the Extracellular Matrix(*p*.adj = 4.36E-09), Collagen Formation(*p*.adj = 1.26E-09) and Collagen Degradation(*p*.adj = 3.78E-10) were most enriched in GSE10667 (S2 Fig 2B). The above results indicate that there is obvious fibroproliferation during the progression of both PDR and IPF. GSEA revealed that although both have a fibrotic core, their pathway activation focuses are different. The PDR dataset is more enriched in angiogenesis and cytoskeletal dynamics – related pathways, while the IPF dataset more prominently shows cell proliferation cycles and ECM remodeling. This may reflect the differences in the pathological structures specific to different tissues. PDR is characterized by the retinal neovascular complex, and IPF is characterized by fibroblast foci.

To conduct the functional enrichment analysis of DEGs2, we performed GO and KEGG pathway enrichment analyses on DEGs2. The GO analysis showed significant enrichment in three major categories. In biological processes, significant enrichments were noted in the organization of external encapsulating structures, extracellular structures, extracellular matrix remodeling, and collagen fibril assembly. For cellular components, the predominant enrichments were in collagen-related structures such as extracellular matrices, trimeric fibrillar collagen, and collagen trimers. In the molecular function (MF) category, genes were predominantly enriched in extracellular matrix structural components, especially those involved in maintaining mechanical strength and cellular adhesion via integrin binding (S2 Fig 2D). Moreover, KEGG pathway analysis showed significant enrichment in biological processes such as protein digestion and absorption, responses to pathogenic infections, and extracellular matrix-receptor signaling interactions (S2 Fig 2C). Together, these findings strongly suggest the activation of fibrotic mechanisms during the progression of both proliferative diabetic retinopathy and idiopathic pulmonary fibrosis.

### Analysis of protein-protein interaction networks and identification of key hub genes

The PPI network of differentially expressed genes (DEGs) was constructed using the STRING database and visualized with Cytoscape (Fig 2A). For hub gene identification, we implemented three complementary computational approaches. Specifically, first, we used the MCODE plugin in Cytoscape to extract the most significant network module, and all constituent genes were considered as potential hub genes (Fig 2D). Second, the cytoHubba plugin’s maximal clique centrality (MCC) algorithm identified 15 candidate genes (Fig 2B). Third, the network centrality analysis (NCA) plugin selected another 15 genes based on unweighted degree centrality (Fig 2C). We used Venn diagrams to perform an intersection analysis of these three independent methods (Fig 2E). Through this analysis, we ultimately identified thirteen consensus hub genes: *COL1A1, COL1A2, THBS2, COL3A1, COL5A1, COL5A2, COL6A2, BGN, COL15A1, COL4A2, POSTN, COL6A1* and *FBN1*.

### Evaluation of the expression of eight hub genes for verification and diagnostic purposes

**Fig 3:**
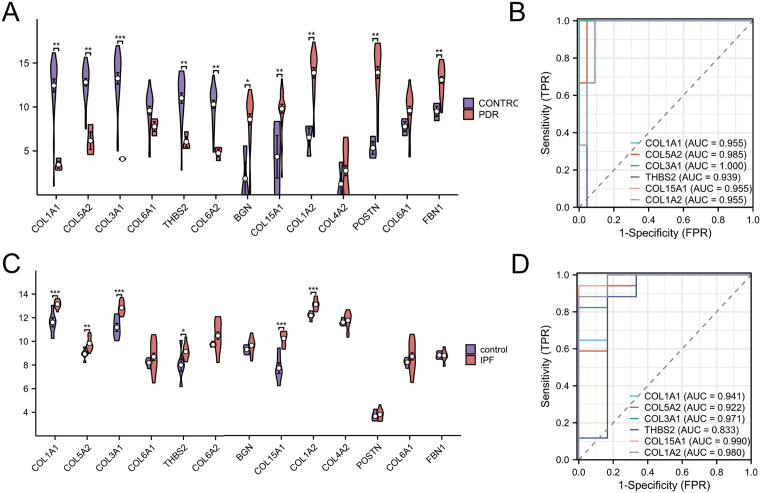
Validation of hub genes and ROC analysis with GEO datasets. **(A)** Transcriptional profiles of the 13 candidate hub genes were analyzed in the GSE102485 dataset for PDR. **(B)** ROC analysis was conducted to assess the diagnostic potential of six selected hub genes in PDR. **(C)** Expression patterns of the 13 hub genes were examined in the GSE24206 dataset for IPF. **(D)** An ROC curve analysis was performed to evaluate the diagnostic accuracy of the six primary hub genes in IPF. *AUC* values denote the area under the ROC curve. Statistical significance was indicated by: ***p < 0.001, **p < 0.01, *p < 0.05.

To validate the identified hub genes’ expression patterns and diagnostic potential, we retrieved two independent datasets (GSE102485 for PDR and GSE24206 for IPF) from the GEO database (S6 and S7 Tables). Using the ggplot2, stats and car packages in R, we generated comparative expression profiles by visualizing them as box-plots. Our analysis revealed differential expression patterns across the datasets: in PDR samples, ten collagen-related genes (*COL1A1, COL5A2, COL3A1, THBS2, COL6A2, BGN, COL15A1, COL1A2, POSTN* and *FBN1*) showed significant upregulation compared to controls (Fig 3A). Similarly, six genes (*COL1A1, COL5A2, COL3A1, THBS2, COL15A1* and *COL1A2*) exhibited elevated expression in IPF samples (Fig 3C). Therefore, these six overlapping genes were selected for subsequent diagnostic evaluation.

To assess the discriminatory power of these candidate biomarkers, we performed ROC curve analysis. In PDR, the *AUC* values demonstrated excellent diagnostic performance: *COL1A1*(*AUC*:0.955), *COL5A2*(*AUC*:0.985), *COL3A1*(*AUC*:1.000), *THBS2*(*AUC*:0.939), *COL15A1*(*AUC*:0.955) and *COL1A2*(*AUC*:0.955) (Fig 3B). The IPF dataset yielded similarly robust results, with the AUC for *COL1A1, COL5A2, COL3A1, THBS2* and *COL15A1* were 0.941,0.922,0.971,0.833, 0.990 and 0.980. These findings suggest that the six collagen-related genes (*COL3A1, COL5A2, COL1A1, THBS2, COL15A1, COL1A2*) may serve as valuable molecular markers for distinguishing between PDR and IPF pathological conditions related to fibrosis (Fig 3D). The consistent overexpression patterns and high diagnostic accuracy across both datasets bolster their potential clinical utility.

### Infiltration of immune cells and the relationship between hub genes and immune cells

**Fig 4:**
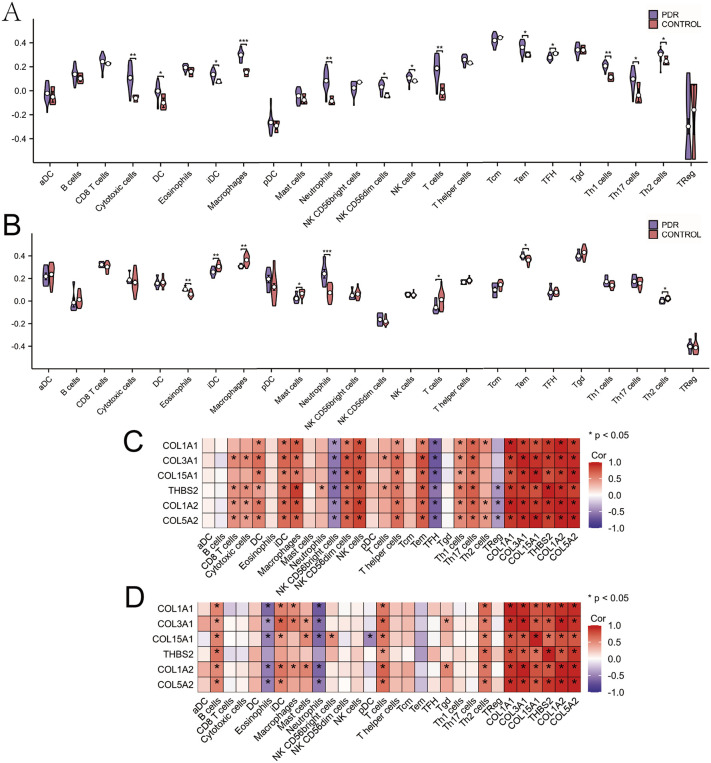
Immunological profiling and hub gene-immune cell interactions. **(A)** Comparative analysis of immune cell composition in GSE102485 reveals differential expression across 24 immune cell subtypes between PDR patients and controls. **(B)** Comparative analysis of immune cell composition in GSE24206 comparing IPF patients with controls. **(C)** Associations between hub genes and 24 immune cell populations in GSE102485. **(D)** Associations between hub genes and 24 immune cell populations in GSE24206.

Emerging research shows that immune cell infiltration and persistent inflammatory responses are involved in the pathogenesis of both proliferative diabetic retinopathy (PDR) and idiopathic pulmonary fibrosis (IPF). To explore this phenomenon, we employed single-sample gene set enrichment analysis (ssGSEA) to quantify immune cell populations. The Mann-Whitney U test was employed for statistical comparisons between groups (S8 and S9 Tables). In the PDR cohort, our analysis showed significant infiltration of multiple immune cell subtypes, such as cytotoxic lymphocytes, dendritic cells (conventional and immature), macrophages, neutrophils, natural killer cells (CD56dim and general), and various T cell subsets (effector memory, follicular helper, Th1, Th17, and Th2) (Fig 4A). The IPF dataset also showed notable involvement of immune cells, particularly eosinophils, immature dendritic cells, macrophages, mast cells, neutrophils, and specific T cell populations, including effector memory and Th2 subsets (Fig 4B). Following correlation analysis between immune cell profiles and six key hub genes provided important insights. In PDR samples, the six hub genes showed a strong positive correlation with various immune cell types, including both mature and immature dendritic cells, macrophages, subsets of natural killer cells (CD56dim and general NK cells), and multiple T-helper cell lineages (Th1 and Th17) (Fig 4C). In IPF specimens, the hub genes showed significant positive correlations with B lymphocytes, total T cells, and Th2 cells (Fig 4D). These findings indicate that the immune – inflammatory patterns of the two diseases may be similar. PDR tends to present a chronic pro – inflammatory microenvironment driven by Th1/Th17. Conversely, IPF may show a stronger Th2 response and B cell – mediated humoral immunity, which contributes to fibrosis formation. Moreover, hub genes may provide specific homing or activation signals for these immune cells by modulating the ECM..

### Transcription factor interaction analysis

**Fig 5:**
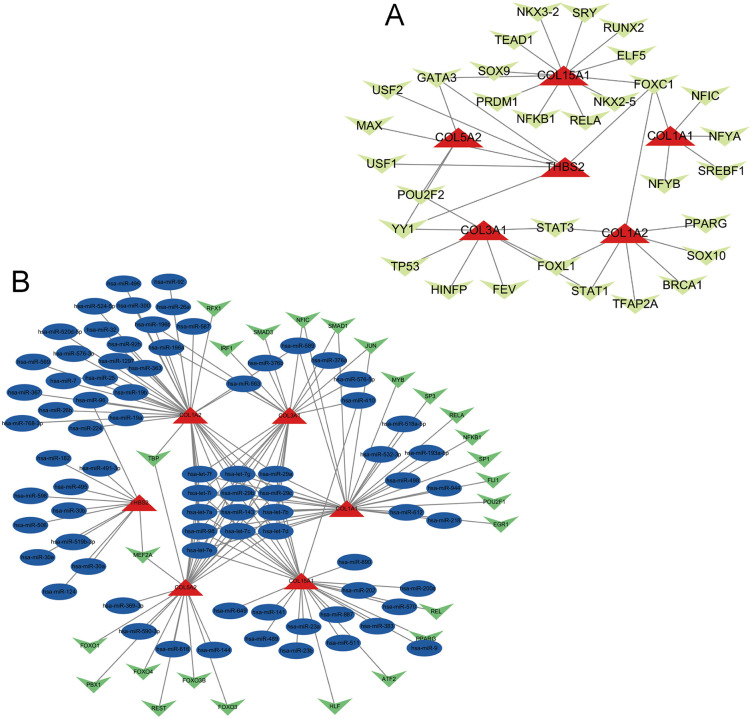
Gene regulatory networks. **(A)** Transcriptional regulatory network illustrating interactions between transcription factors and hub genes. **(B)** Post-transcriptional regulatory network depicting miRNA-mediated regulation of hub genes.

To elucidate the transcriptional regulatory mechanisms of hub genes, we used NetworkAnalyst to construct and visualize transcription factor (TF)-gene interaction networks. The primary TF-gene network had 37 molecular nodes. These nodes were interconnected by 42 regulatory edges. Analysis with the JASPAR database revealed 31 potential transcriptional regulators, with seven key TFs (*FOXC1, GATA3, YY1, FOXL1, STAT3, STAT1* and *POU2F2*), as illustrated in Fig 5A, demonstrating significant regulatory connectivity (degree ≥ 2). However, these predictions require experimental confirmation.

Furthermore, we established an expanded regulatory network that included six hub genes, 77 microRNAs, and 26 transcription factors. This network had a complex interaction landscape with 109 edges connecting 162 nodes (Fig 5B). Network topology analysis identified *COL1A1* as the most highly connected node (degree = 40); *COL5A2* (degree = 25) and *COL3A1* (degree = 24) followed; both *THBS2* and *COL15A1* exhibited moderate connectivity (degree = 12).

### Identification of therapeutic small molecules targeting key hubs

Using the Connectivity Map (cMAP) database for comprehensive computational analysis, we found promising small-molecule candidates targeting the common hub genes systematically. Our screening approach gave priority to compounds showing the most significant enrichment scores, ultimately selecting six potential therapeutic agents: bruceantin, BRD-K76674262, BRD-K24017250, SB-431542, BRD-K67506692, and panobinostat. These molecules show considerable potential for anti-fibrotic therapy in both proliferative diabetic retinopathy (PDR) and idiopathic pulmonary fibrosis (IPF). Their three-dimensional molecular structures are illustrated in S3 Fig.

**Fig 6:**
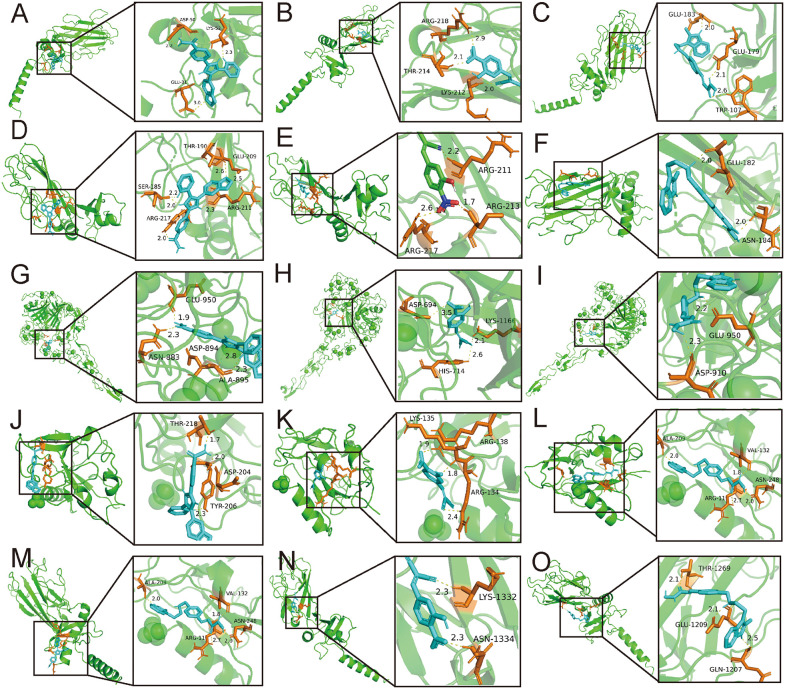
Molecular docking simulation results demonstrating ligand-receptor interactions. **(A)** SB-431542 established three hydrogen bonds with *COL1A1* residues, with interatomic distances of 2.2 Å, 2.3 Å, and 3.0 Å. **(B)** BRD-K7506692 formed three hydrogen bonds with *COL1A1*, with bond lengths of 2.0 Å, 2.1 Å, and 2.9 Å. **(C)** Panobinostat formed two hydrogen bonds with *COL1A1* residues, measuring 2.0 Å, 2.1 Å and 2.6 Å. **(D)** *COL3A1* formed hydrogen bonds with SB-431542 (2.0 Å, 2.0 Å, 2.2 Å, 2.3 Å, 2.5 Å and 2.6 Å). **(E)** BRD-K7506692 formed two hydrogen bonds with *COL3A1* (1.7 Å, 2.2 Å, 2.6 Å). **(F)** Panobinostat formed three hydrogen bonds with *COL3A1*(2.0 Å, 2.0 Å). **(G)** *THBS2* interacted with SB-431542 (1.9 Å, 2.3 Å,2.3 Å, 2.8 Å). **(H)** *THBS2* interacted with BRD-K7506692 (2.1 Å, 2.6 Å, 3.5 Å). **(I)** *THBS2* formed bonds with Panobinostat (2.2 Å, 2.3 Å). **(J)** *COL15A1* bonded with SB-431542 (1.7 Å, 2.0 Å, 2.3 Å). **(K)** *COL15A1* formed bonds with BRD-K7506692 (1.8 Å, 1.9 Å, 2.4 Å). **(L)** *COL15A1* interacted with Panobinostat (1.8 Å, 2.0 Å, 2.0 Å, 2.7 Å). **(M)** *COL15A2* established four hydrogen bonds with SB-431542 (2.1 Å, 2.2 Å, 2.3 Å, 2.6 Å). **(N)** *COL15A2* formed bonds with BRD-K7506692 (2.3 Å, 2.3 Å). **(O)** Panobinostat interacted with *COL15A2* through bonds measuring 2.1 Å, 2.1 Å and 2.5 Å.

To elucidate potential therapeutic mechanisms, we performed molecular docking simulations to assess the binding affinities between these compounds and five hub genes (excluding *COL5A2* because the structural data were unavailable). The docking results, presented in Table 2, showed better binding energies for SB-431542, BRD-K67506692 and panobinostat compared to the other candidates. Therefore, this enhanced binding affinity suggests that these three compounds may exhibit particularly potent anti-fibrotic effects in PDR and IPF treatment.Detailed molecular interactions are visualized in Fig 6, providing structural insights into the potential targets for therapy.

**Table 2:** The result of molecular docking.

Drug molecules	PubChem ID	Docking Score(kcal/mol)				
		*COL1A1*	*COL3A1*	*THBS2*	*COL15A1*	*COL1A2*
burceantin	5281304	−4.68	−3.18	−5.1	−5.66	−3.84
BRD-K76674262	285033	−3.58	−1.04	−2.54	−4.46	−0.92
BRD-K24017250	54639109	−3.15	−4.84	−2.55	−4.65	−3.27
SB-431542	4521392	−5.56	−7.34	−5.79	−7.15	−5.48
BRD-K67506692	2046	−5.54	−5.59	−4.69	−6.43	−5.01
panobinostat	6918837	−4.25	−6.13	−5.64	−8.49	−4.03

## Discussion

In this study, bioinformatics analysis methods were used to search fibrosis-related datasets of PDR and IPF, and 90 common DEGs between PDR and IPF were identified from the GEO database. GSEA results revealed that PDR-related datasets were predominantly enriched in growth factor receptor signal transduction, VEGFA-VEGFR2 signaling, *RHO* GTPase cycle, and actin cytoskeleton regulation pathways. The IPF-related datasets showed significant enrichment in processes such as the mitotic cell cycle, extracellular matrix degradation, and both collagen formation and degradation. It indicates that there is obvious fibroproliferation during the progression in both of PDR and IPF. GO enrichment analysis revealed that DEGs were predominantly associated with extracellular structure organization, collagen-containing extracellular matrix, and extracellular matrix structural components.Enriched KEGG pathways comprised protein digestion and absorption, amoebiasis, and ECM-receptor interaction. This suggests that the shared DEGs are significantly associated with the extracellular matrix, metabolism, and immune response.

A PPI network was developed utilizing the STRING database and Cytoscape software. Then, according to the MCODE, cytoNCA, and cytoHubba plug-ins of Cytoscape, we screened 13 hub genes (*COL1A1, COL1A2, THBS2, CLO3A1, COL5A1, COL6A2, BGN, COL15A1, COL1A2, COL4A2, POSTN, COL6A1* and *FBN1*). We assessed the expression of 13 hub genes in validation datasets, aiming to confirm their association with PDR and IPF. Subsequently, we validated the hub genes by combining ROC curve analysis with their expression levels and finally screened six hub genes, namely *COL1A1, COL5A2, COL3A1, THBS2, COL15A1* and *COL1A2*. The upregulation of these six genes in both PDR patients and IPF samples indicates their potential significance in the mechanisms underlying PDR and IPF.

The genes *COL1A1, COL1A2, COL3A1, COL5A2* and *COL15A1* encode structural components of the collagen superfamily. These components mainly reside in the extracellular matrix (ECM) and have characteristic triple-helical domains [25]. Abnormal regulation of collagen biosynthesis has been implicated in various pathological conditions, particularly fibrotic disorders where an imbalance between collagen production and breakdown leads to abnormal ECM accumulation [26,27]. Type I collagen constitutes over 90% of the total collagen content in most tissues, serving as the primary component of the extracellular matrix (ECM) [28]. In pulmonary fibrosis, increased *COL1A1* expression in alveolar macrophages is associated with disease progression [29]. This finding suggests a potential mechanistic link between collagen dysregulation and fibrotic pathogenesis. Previous research has shown that circ*COL1A2* expression is significantly upregulated, with an approximate 6.85-fold increase in diabetic retinopathy patients compared to non-diabetic controls [30]. Furthermore, this circular RNA was found to promote angiogenesis through its regulatory effect on the miR-29b/*VEGF* signaling pathway in diabetic retinopathy pathogenesis [31]. *COL3A1*, a member of the type III collagen family, is co-expressed with type I collagen in various soft connective tissues. This fibrillar collagen serves as a major structural element in hollow visceral organs including major blood vessels, the gastrointestinal tract, and the uterine wall. Notably, in addition to its structural role, *COL3A1* has been implicated in multiple fibrotic disorders characterized by pathological deposition of type III collagen in affected tissues [32]. *COL15A1* is a structural protein that stabilizes microvessels and muscle cells in both the heart and skeletal muscle.Insights gained from *COL15A1 +* endothelial cells (ECs) in one disease context may enhance the understanding of other diseases. The transcriptomic resemblance between NSCLC ‘tip cells’ and *COL15A1* + endothelial cells (ECs) in interstitial lung disease suggests potential angiogenic capabilities for the *COL15A1* + ECs [33,34]. Furthermore, the aforementioned four collagen-related genes are associated with gastroesophageal reflux disease and oesophageal carcinoma and play a role in regulating the immunosuppressive microenvironment [35–37]. *THBS2* is an adhesive glycoprotein facilitating cell-to-cell and cell-to-matrix interactions, acting as a CD36 ligand with antiangiogenic effects. *THBS2* influences angiogenesis, cell motility, apoptosis, and cytoskeletal organization by interacting with extracellular matrix proteins and cell surface receptors [38–40].

Analysis of the six core genes revealed that *COL1A1, COL3A1, COL1A2, COL5A2* and *COL15A1* are primarily associated with fibrosis and collagen metabolism. In contrast, *THBS2* overexpression primarily enhances anti-neoangiogenesis and influences cell adhesion and migration. PDR and IPF development involves pathological processes such as fibrosis, neoangiogenesis, cell adhesion and migration, tissue repair, and immune pro-inflammatory responses. The six screened core genes reflected these processes and summarized their key aspects. These hub genes have been identified in independent datasets on PDR and IPF. This study is the first to screen and integrate six core genes, providing genetic evidence for the shared pathological basis of PDR and IPF.

*Hsa-let-7*, a non-coding small RNA, is crucial for cell growth, apoptosis, and tumor development. The miRNA *hsa-let-7* is recognized as a cancer suppressor [41], known to inhibit lung cancer cell proliferation and activity, and regulate the progression of liver cancer [42], retinoblastoma [43], colon cancer [44], osteosarcoma [45], and other tumors. The study’s TF-miRNA interaction analysis reveals significant involvement of the *let-7* family within the coregulatory network. The *let-7* family (*hsa-let-7a, hsa-let-7b, hsa-let-7c, hsa-let-7e, hsa-let-7f, hsa-let-7g,* and *hsa-let-7i*) exhibited a degree value of four in the network, suggesting their potential significance in future research on PDR and IPF pathogenesis.

Recent studies have highlighted the critical role of inflammation and immunity in the progression of PDR and IPF [46,47]. Neutrophils, T cells, and macrophages, which are innate immune cells, play a crucial role in fibrosis development through immune inflammation. Key substances involved in this immunoinflammatory process include IL-1β, IL-6, transforming growth factor (*TGF*)-β, and tumor necrosis factor (*TNF*)-α [48–50]. In individuals with IPF, there is a significant increase in inflammatory cells such as macrophages, antigen-activated T and B cells, mast cells, and lymphocytes in both the circulation and fibrotic lungs. Meanwhile, the activation of macrophages, neutrophils, and T cells in patients’ whole body or local eyes plays significant roles in DR [51]. Evidence shows that the activation, polarization, and function of macrophages, neutrophils, and T cells are crucial in the diabetes-induced inflammatory reaction [52]. Our analysis of immune cells in PDR and IPF patient samples corroborates the findings on immune cells’ involvement in diabetes-related inflammation. We also analyzed immune cell infiltration near *COL1A1, COL3A1, COL1A2, COL15A1, COL5A2* and *THBS2* in PDR and IPF patients, which is pertinent to disease progression. In PDR samples, a strong positive correlation was observed between the 6 hub genes and various immune cells, including DC cells, iDC cells, Macrophages, NKCD56dim cells, NK cells, T-helper cells, Tem cells, Th1 cells, and Th17 cells. There was a notable positive association observed between B cells, T cells, and Th2 cells and the 6 hub genes in the IPF samples. The positive associations between immune cells and hub genes in PDR and IPF samples further confirm the involvement of macrophages, neutrophils, and T cells in the progression of PDR and IPF fibrosis.

Integrating prior relevant research findings with our analyses, we have attempted to reconstruct the fibrotic progression processes of both PDR and IPF. To begin with, *THBS2* activates latent TGF – β by direct binding, releasing active TGF – β, the most potent fibrosis inducer [53,54]. Subsequently, active *TGF-β* initiates a fibrotic cascade characterized by the enhanced expression of *COL1A1*, *COL1A2*, *COL3A1*, and *COL5A2*, whose products (types I, III, and V collagens) are secreted into the extracellular space [55]. Once secreted into the extracellular space, types I, III, and V collagens perform distinct roles: type I confers tensile strength, type III enhances elasticity, and type V regulates fibril assembly and diamete [56]. As fibrosis progresses, increased collagen cross-linking elevates tissue rigidity, a mechanical change that is detected by cellular mechanoreceptors. In turn, this mechanotransduction event activates downstream profibrotic signaling cascades, including *YAP/TAZ* and *Rho* GTPase, which potentiate *TGF-β* activity, establishing a self-reinforcing feedback loop that drives progressive fibrosis [57]. Beyond these mechanical effects, this aberrant ECM network shapes a pro-fibrotic immune microenvironment via diverse mechanisms. For instance, increased accumulation of collagen and *THBS2* promotes immune cell adhesion, infiltration, and retention, while concurrently skewing immune cell phenotypes toward a pro-fibrotic state [58]. Additionally, matrix metalloproteinases (*MMPs*) degrade the ECM, generating damage-associated molecular patterns (DAMPs) that recruit and activate immune cells. At the same time, *COL15A1* drives dysregulated neovascularization, which results in plasma protein extravasation and subsequent tissue hypoxia [59]. Collectively, recruited immune cells secrete pro – fibrotic cytokines and growth factors,thereby amplifying the fibrosis cycle [60]. Taken together, these six genes constitute a self-reinforcing pro-fibrotic circuit. In both PDR and IPF, they serve as master regulators that couple inflammatory cues, mechanical strain, and ECM rigidity into a progressive pathological cascade [61]. Consequently, this panel of six core genes serves as a valuable auxiliary computational biology tool, offering critical insights into disease diagnosis, assessment, staging, and monitoring. Furthermore, these genes represent promising targets for the development of multi-target therapeutic strategies against PDR and IPF [62].

In summary, we identified common differentially expressed genes (DEGs) and isolated six hub genes shared between PDR and IPF. Utilizing the six hub genes, we constructed a TF-miRNA coregulatory network and identified potential drug molecules. In the study, we selected six drug molecules from all the candidate drugs. SB-431542, BRD-K67506692 and panobinostat had higher binding energies by the analysis of autodock. SB-431542 selectively inhibits transforming growth factor beta (*TGF-β*) receptor type I (*ALK5*), as well as activin receptor-like kinases 4 (*ALK4*) and 7 (*ALK7*) [63–65]. Panobinostat is a novel histone deacetylase (*HDAC*) inhibitor that exerts anti-tumor effects by regulating gene expression [64–66]. The function and mechanism of BRD - k67506692 require further exploration. These three drugs show potential in the anti-fibrotic treatment of PDR and IPF by affecting cell proliferation and differentiation.

However, future experimental and clinical studies are essential to validate these findings, as our conclusions are primarily based on computational analyses without direct wet-lab validation. Specifically, validation will include: (1) immunohistochemical (IHC) detection of key proteins (e.g., COL3A1, THBS2) in clinical samples from PDR (i.e., fibrovascular membranes) and IPF (i.e., lung tissues); (2) gain- and loss-of-function assays in relevant in vitro models (human retinal microvascular endothelial cells for PDR and human lung fibroblasts for IPF) to assess gene roles in proliferation, migration, and extracellular matrix (ECM) secretion; and (3) evaluation of potential therapeutic targets in PDR and IPF animal models. Through these experiments, we aim to validate our findings and assess their translational potential.

## Conclusions

Our research is subject to three limitations. First, we utilized data extracted from a publicly accessible database, which had specific constraints related to the sample size. Secondly, accessing clinical information from public databases is inefficient. Third, insufficient evidence shows that *COL1A1, COL1A3, COL15A1, COL1A2, COL5A2, THBS2* are powerful diagnostic markers for PDR and IPF patients. Future experimental and clinical studies are essential to validate these findings.

## Supporting information

S1 Table Fibrosis-related genes from the GeneCards database. (XLSX)

S2 Table Differential analysis results of GSE94019. (XLSX)

S3 Table Differential analysis results of GSE10667. (XLSX)

S4 Table The intersection of differential genes between GSE94019 and GSE10667. (XLSX)

S5 Table Intersection of fibrosis-related genes and differentially expressed genes in PDR and IPF. (XLSX)

S6 Table Hub genes identification in GSE102485: results of group comparison using Mann-Whitney U test. (XLSX)

S7 Table Hub genes identification in GSE24206: results of group comparison using Mann-Whitney U test. (XLSX)

S8 Table Identification of 24 immune cells in GSE102485: results of group comparison using Mann-Whitney U test. (XLSX)

S9 Table Identification of 24 Immune Cells in GSE24206: Results of Group Comparison using Mann-Whitney U test. (XLSX)

S1 Fig Workflow of this study. (TIF)

S2 Fig Visualization of functional enrichment analysis. (A) The bubble chart of GSEA depicted in Figure lists the top 10 gene sets in GSE94019; (B) The bubble chart of GSEA in GSE10667; (C) The bubble chart of KEGG analysis of DEGs2; (D) The result of GOKEGG analysis of BP, CC and MF.
(TIF)

S3 Fig The three-dimensional structure of six molecules. (A) Bruceantin; (B) BRD-K76674262; (C) BRD-K24017250; (D) SB-431542; (E) BRD-K67506692; (F) panobinostat.
(TIF)
