# Correction: Integrated bioinformatics-based identification of proliferative diabetic retinopathy and idiopathic pulmonary fibrosis: Focus on fibrosis and immune infiltration

**DOI:** 10.1371/journal.pone.0345652

**Published:** 2026-03-23

**Authors:** Juanhui Cao, Fangyuan Dong, Xianfeng Li, Xuechun Zhu

In the Discussion section, there is an error in the second sentence of the seventh paragraph. The correct sentence is: To begin with, THBS1 activates latent TGF-β by direct binding, releasing active TGF-β, the most potent fibrosis inducer [53,54].
