# Supplementary figures and images for: Integrated bioinformatics-based identification of proliferative diabetic retinopathy and idiopathic pulmonary fibrosis: Focus on fibrosis and immune infiltration
Source: PLoS One. 2026 Feb 23;21(2):e0343398. doi: 10.1371/journal.pone.0343398 (PMC12928582; doi:10.1371/journal.pone.0343398)

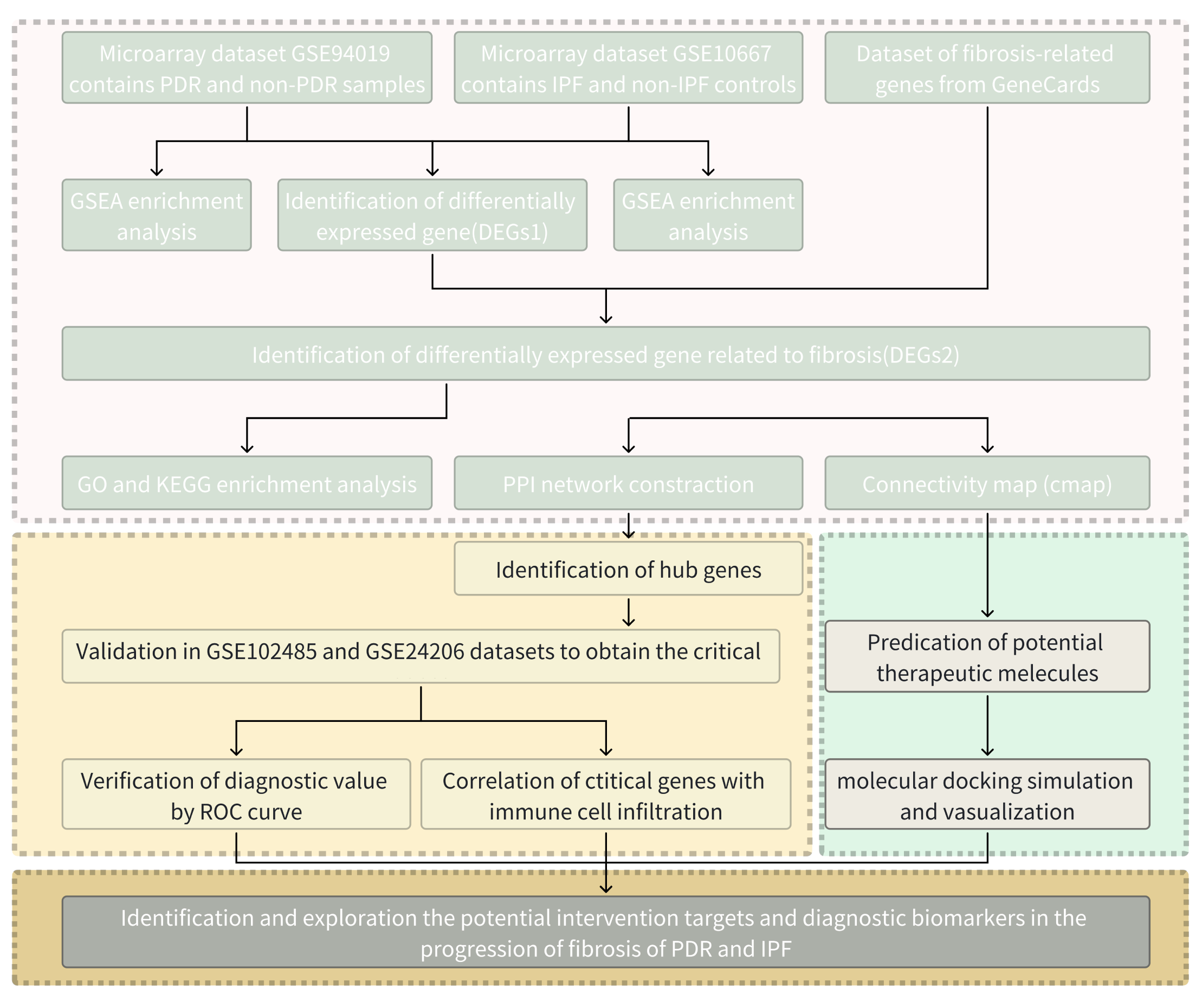

Supplement: S1 Fig — (TIF) [file pone.0343398.s010.tif]

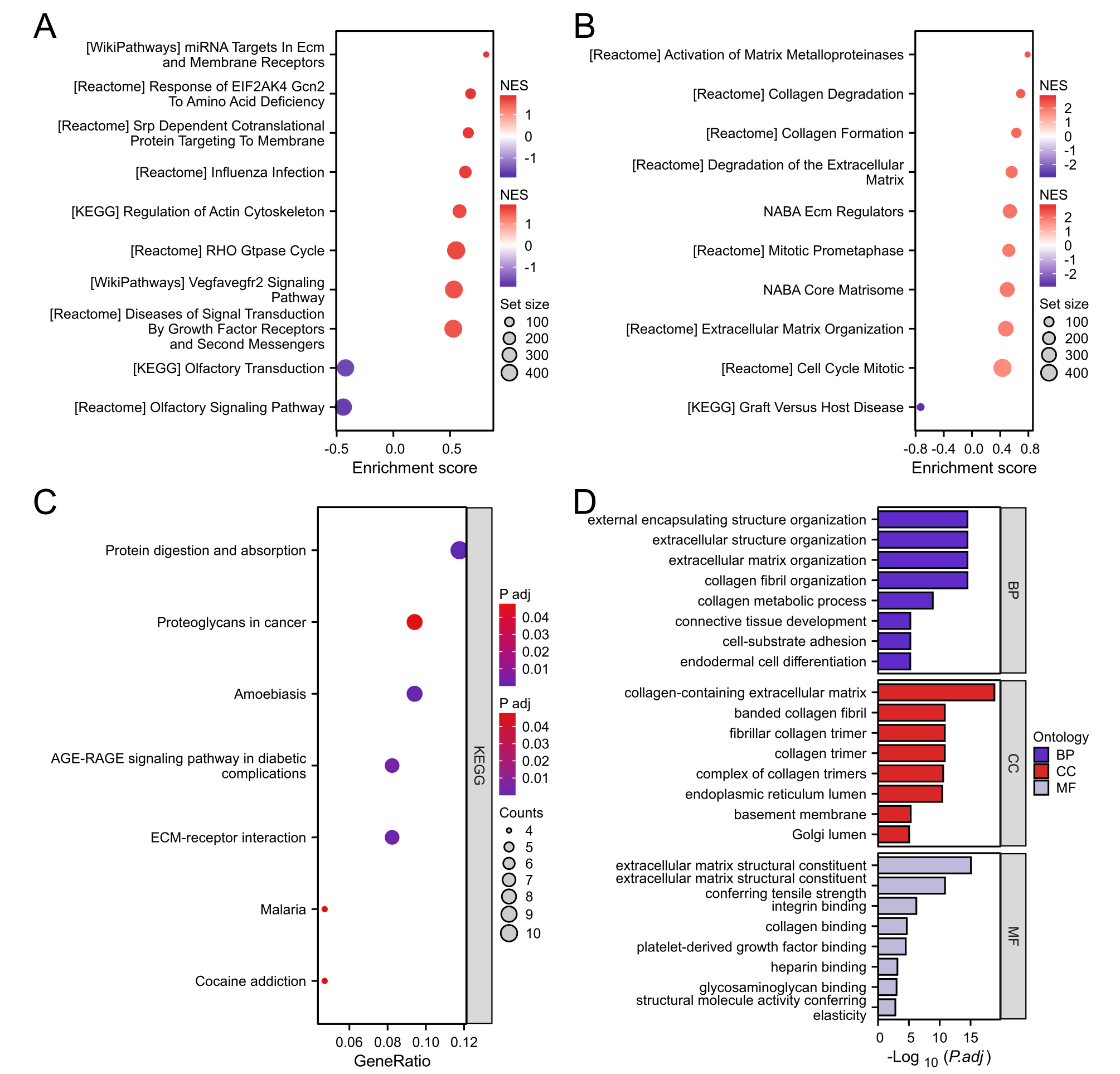

Supplement: S2 Fig — (A) The bubble chart of GSEA depicted in Figure lists the top 10 gene sets in GSE94019; (B) The bubble chart of GSEA in GSE10667; (C) The bubble chart of KEGG analysis of DEGs2; (D) The result of GOKEGG analysis of BP, CC and MF. (TIF) [file pone.0343398.s011.tif]

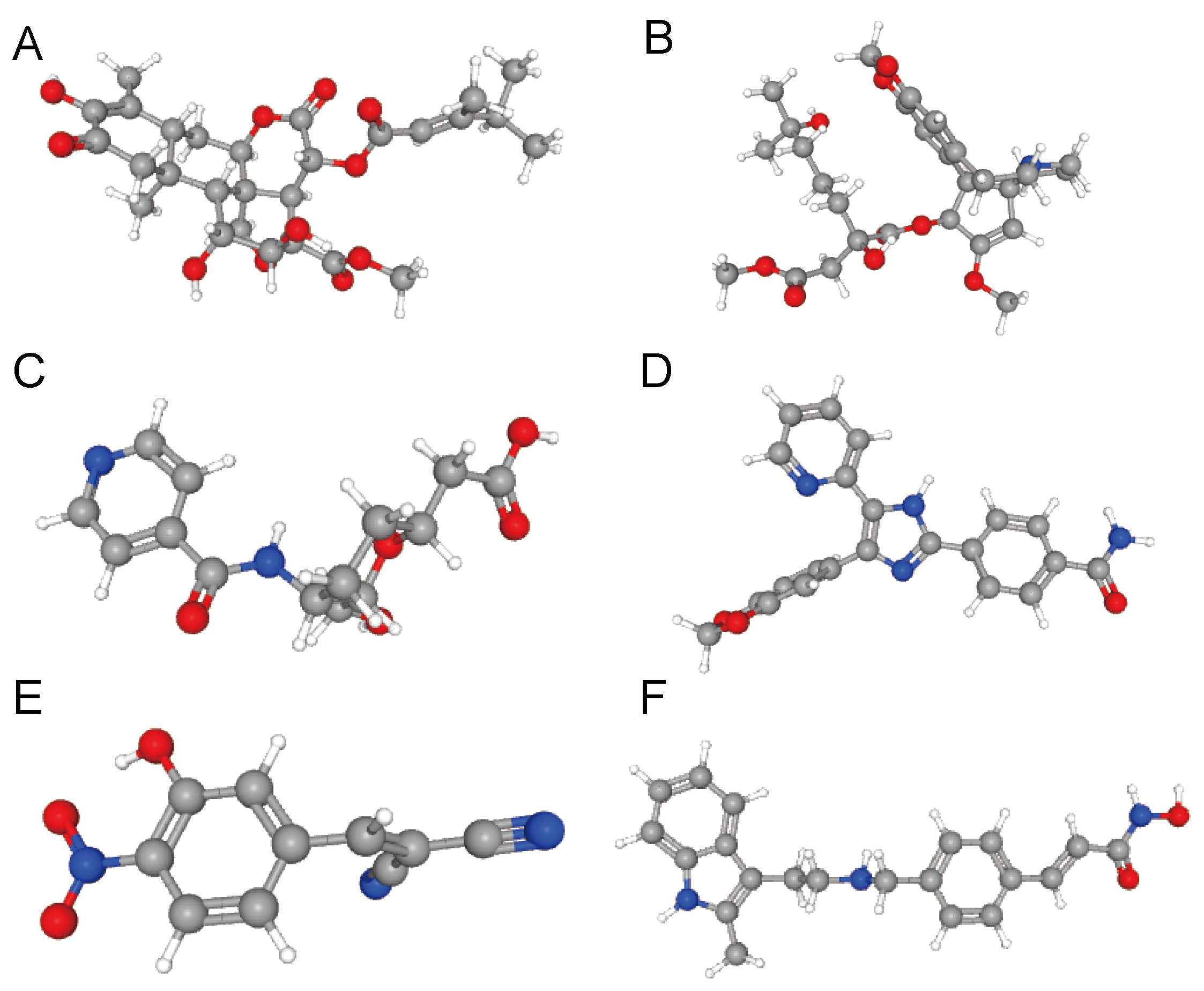

Supplement: S3 Fig — (A) Bruceantin; (B) BRD-K76674262; (C) BRD-K24017250; (D) SB-431542; (E) BRD-K67506692; (F) panobinostat. (TIF) [file pone.0343398.s012.tif]
